# Gingival Cyst of the Adult as Early Sequela of Connective Tissue Grafting

**DOI:** 10.1155/2015/473689

**Published:** 2015-07-06

**Authors:** Mariana Gil Escalante, Dimitris N. Tatakis

**Affiliations:** ^1^Division of Periodontology, College of Dentistry, The Ohio State University, Columbus, OH 43210, USA; ^2^Private Practice, San Jose, Costa Rica

## Abstract

The subepithelial connective tissue graft (SCTG) is a highly predictable procedure with low complication rate. The reported early complications consist of typical postsurgical sequelae, such as pain and swelling. This case report describes the development and management of a gingival cyst following SCTG to obtain root coverage. Three weeks after SCTG procedure, a slightly raised, indurated, ~5 mm diameter asymptomatic lesion was evident. Excisional biopsy was performed and the histopathological evaluation confirmed the gingival cyst diagnosis. At the 1-year follow-up, the site had complete root coverage and normal tissue appearance and the patient remained asymptomatic.

## 1. Introduction

The subepithelial connective tissue graft (SCTG) procedure, first introduced for root coverage in 1985 [1, 2], is considered the gold standard for treatment of gingival recession defects where complete root coverage and gain of keratinized tissue are the desired outcomes [3]. Although SCTG is a commonly used procedure, the number and prevalence of reported postoperative complications, which have been characterized as either early (days to few weeks) or late (few months to years), are limited [4–6]. Since the procedure involves two intraoral surgical sites (palatal donor site and recipient site), postoperative complications may occur on either of them. Early complications relating to the donor site include pain [4, 5, 7, 8] and bleeding [4, 5] and more rarely necrosis of the palatal overlying tissue [9–11]. Regarding the recipient site, the most commonly reported early complications include pain [4, 5, 7] and swelling [4, 5, 12]. Other early complications in the recipient site include bleeding, sensitivity, ecchymosis, loose sutures, and poor graft immobilization [4]. Although the literature contains a few reports of late SCTG complications that required histopathological assessment [6], early complications that require biopsy for definitive assessment have not been reported.

This report presents a hitherto unreported early complication of SCTG, namely the development of a gingival cyst of the adult (GCA), describes the management of this complication and reviews similar postoperative sequelae.

## 2. Case Report

**Figure 1:**
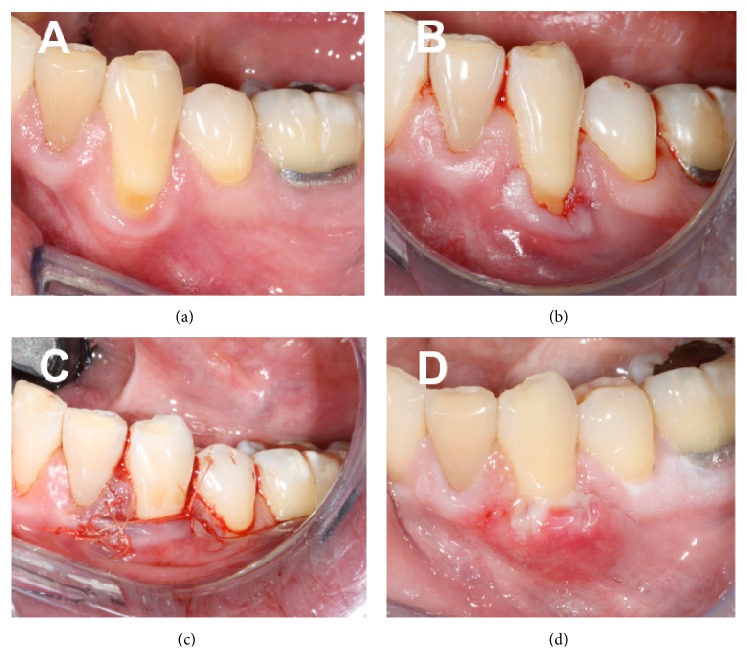
Clinical images. (a) Initial presentation; note Miller Class I recession defect on facial of the mandibular left canine; (b) pouch prepared; (c) SCTG surgery completed; (d) postoperative week 1 presentation.

A 46-year-old African American male was referred by the Dental Student clinic to the Graduate Periodontology clinic at the Ohio State University for treatment of gingival recession on the facial aspect of the mandibular left canine. Clinical examination revealed approximately 3 mm of gingival recession (Miller Class I) and less than 1 mm of attached gingiva on the tooth (Figure 1(a)).

The patient's medical history was unremarkable and his periodontal diagnosis was plaque-induced gingivitis, for which he had received prophylaxis in the Dental Student clinic. After the consultation appointment, the patient was scheduled for the SCTG procedure. Under local anesthesia, and following thorough root planing of the exposed root surface, a full thickness envelope flap (pouch) was elevated on the affected area (Figure 1(b)). The SCTG graft was harvested from the ipsilateral palatal premolar area using a parallel incision technique. The palatal harvest site was sutured with 4-0 polyglycolic acid (PGA) sutures. The graft was transplanted to the prepared donor site and secured with 4-0 PGA sutures. The graft was partially covered with the overlaying flap, and the flap was secured to the undisturbed interproximal papillae with 4-0 PGA simple interrupted sutures (Figure 1(c)). Hemostasis was achieved on both donor and recipient sites and the patient was dismissed with no complications. Postoperative instructions included no mechanical plaque control in the area for at least 3 weeks, soft diet for the first week, and avoidance of trauma to either surgical site. The patient was given prescriptions for analgesic/anti-inflammatory medication (ibuprofen 600 mg, TID) and for antimicrobial rinse (Chlorhexidine gluconate 0.12%, BID).

**Figure 2:**
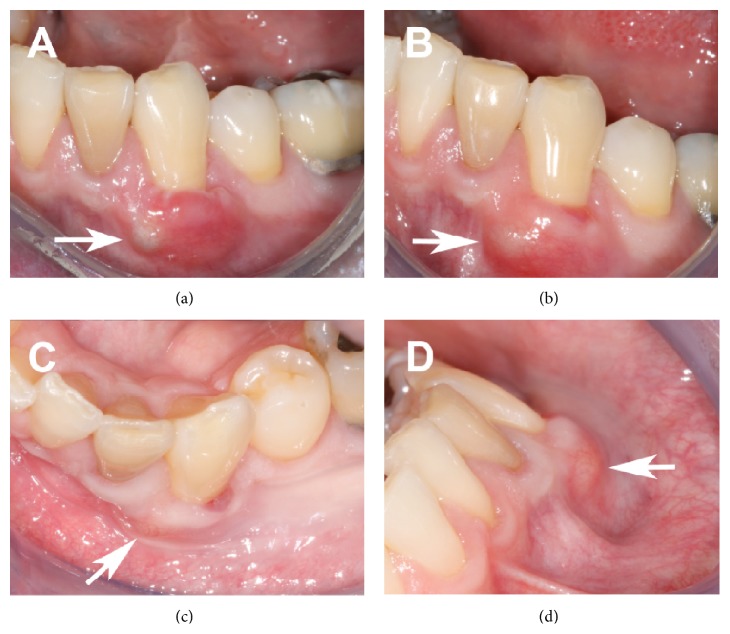
Clinical images. (a) Postoperative week 3 presentation; note lesion on mesial aspect of treated recession defect (arrow); (b–d) postoperative week 6 presentation; lesion (arrow) evident from direct (b), occlusal (c), and profile (d) view.

Clinical presentation at the first postoperative visit (1 week) was within normal limits and the patient reported no pain or swelling (Figure 1(d)). At the 3-week postoperative visit, an asymptomatic, slightly indurated, 5 mm swelling was noted on the mesioapical corner of the recipient site (Figure 2(a)).

**Figure 3:**
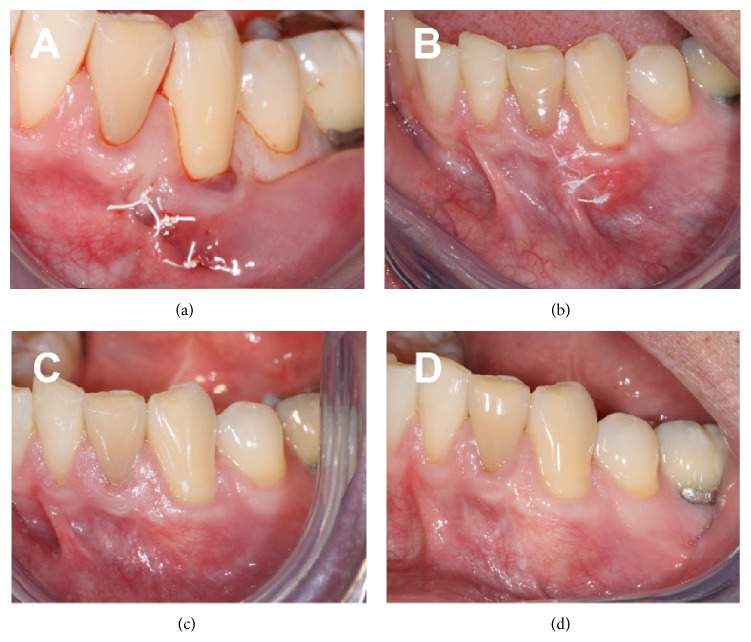
Clinical images. (a) Excisional biopsy completed; (b–d) postoperative appearance: 1 week (b), 3 months (c), and 12 months (d).

No drainage could be elicited. The overlying tissue was normal in appearance and the treated tooth tested vital. The radiographic appearance of the area was within normal limits and without any change from initial presentation. Since it was still relatively early in the postoperative course, the patient was informed and instructed to return in 3 more weeks. At the 6-week postoperative visit, the asymptomatic lesion was still evident and appeared slightly enlarged from the previous visit (Figures 2(b)–2(d)). Following patient consent, an excisional biopsy was performed under local anesthesia. Immediately upon removal, the 5 × 2 × 5 mm lesion was placed in formalin and sent for histopathological analysis, with “gingival cyst” as the working diagnosis. The biopsy site was sutured using nonabsorbable sutures (Figure 3(a)), to eliminate any possible local irritation from the degradation of absorbable suture material. The biopsy specimen was processed for routine hematoxylin-eosin (H&E) staining.

One week after the biopsy, the patient reported no symptoms and the site was healing within normal limits; sutures were removed (Figure 3(b)). At the 3-month follow-up the patient was still asymptomatic and clinical examination revealed no recurrence of the lesion and complete root coverage (Figure 3(c)). At the 1-year follow-up, the patient remained asymptomatic and reported being satisfied with the outcome of the SCTG procedure. Clinically, complete root coverage with normal appearing tissues was evident (Figure 3(d)).

**Figure 4:**
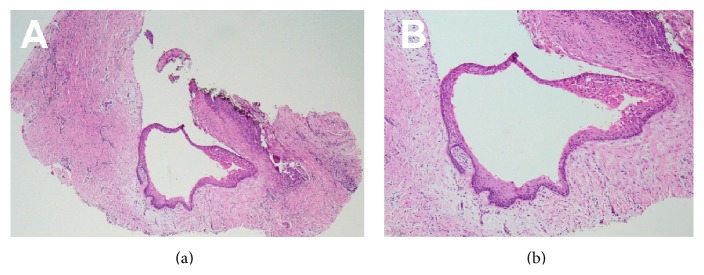
Routine histology of lesion: low-power (a) and higher power (b) photomicrographs. Note the stratified squamous nonkeratinized cystic epithelium lining the lesion and the surrounding subacutely inflamed fibrous connective tissue (hematoxylin and eosin, original magnification: (a) ×40; (b) ×100).

The pathology report provided a description of subacutely inflamed fibrous connective tissue associated with stratified squamous nonkeratinized cystic epithelium and confirmed the diagnosis of gingival cyst (Figure 4).

## 3. Discussion

The present report documents a histopathologically diagnosed gingival cyst that developed within 3 weeks following a SCTG procedure. This is the first case of a histopathologically diagnosed gingival cyst of the adult (GCA) presenting as an early SCTG complication.

The literature on GCA, which was reviewed few years ago [18], indicates that this relatively rare developmental odontogenic cyst is most prevalent in the 5th and 6th decade (mean age at presentation: 49 years), most common in the mandible (80%) and in canine-premolar sites, typically presenting as solitary lesion (76%) of ~5 mm diameter, and is treated by excisional biopsy. The GCA does not have a strong gender or race predilection, with both females and whites accounting for 60% of the cases where gender or race information was provided [18]. The present case characteristics (patient age, jaw, site, and size) are consistent with the typical GCA demographics. The differential diagnosis of GCA includes several lesions presenting as gingival swellings, including odontogenic keratocyst, lateral periodontal cyst, peripheral fibroma, peripheral ossifying fibroma, peripheral giant cell granuloma, pyogenic granuloma, mucocele, parulis, and periapical cyst of endodontic origin [18]. In the present case, the characteristics of the soft tissue presentation, the lack of radiographic findings, and the vitality of the associated tooth together suggested that the lesion was a GCA. GCA recurrence after excision is quite rare [18, 19], and the present case showed no signs of recurrence one year after removal.

Cystic and cyst-like lesions following SCTG [13–15, 17] or free-gingival graft (FGG) [16] procedures have been reported previously; however, all of the reported cases were late complications which manifested 9–48 months postoperatively. The present case is the first one of a histopathologically diagnosed GCA presenting as an early SCTG complication, that is, within the first 3 postoperative weeks after the root coverage procedure. The characteristics of the reported cystic and cyst-like lesions following soft tissue grafting are summarized in Table 1.

Of the six reported cases, all involved anterior teeth and five were located in the mandible. The sole maxillary case was associated with multiple SCTGs performed for ridge augmentation in relation to implant treatment [17]. One case of “cul-de-sac” was diagnosed clinically, as no specimen was available for histopathological diagnosis [15]. All lesions were relatively small (3–6 mm) and three of them presented with some form of discharge [14–16]. None of the lesions appeared to compromise the outcome (root coverage or gingival augmentation) of the soft tissue grafting procedure, although in two cases [15, 16] the clinicians opted to perform a secondary graft following lesion removal. None of the cases had a recurrence during follow-up.

The etiology underlying the development of cystic lesions after soft tissue grafting is unclear. Histologic assessment of healed, asymptomatic, and clinically normal FGGs [20] and SCTGs [21, 22] has revealed the presence of epithelial invaginations and “cyst-like spaces.” These epithelial invaginations, which form between the SCTG and the overlying flap, represent projections of normally structured epithelium and typically are devoid of any associated inflammatory cell infiltrate [21]. Furthermore, 80% of SCTGs prepared for placement have some epithelial remnants, despite attempts to remove the epithelium [23]. Both the commonly found epithelial invaginations and the prevalent epithelial remnants could give rise to cystic lesions; however, the scarcity of reported post-SCTG cysts suggests that this is not a common occurrence. Obviously, the mere presence of epithelial tissue is not enough to give rise to a cyst; an additional stimulus is necessary [16]. In the case of cysts developing after soft tissue grafting, the surgical trauma can be reasonably considered as the stimulating factor [15]. The remarkably fast development of a sizeable cyst after SCTG in the present case, in contrast to the delayed development of other documented cystic lesions following soft tissue grafting, raises the possibility that the surgical trauma stimulated the epithelial proliferation of a persisting dormant dental lamina microcyst [24, 25], giving rise to the observed GCA.

In conclusion, the present case indicates that gingival cyst formation is a possible early complication following a subepithelial connective tissue graft procedure to correct gingival recession. Treatment by conservative surgical excision can result in satisfactory outcomes, that is, uncompromised root coverage and lack of recurrence.

## Figures and Tables

**Table 1:** Cyst and cyst-like lesions as sequelae of soft tissue grafting.

Report	Patient^A^	Jaw, Site	Discharge	Preceding procedure	Time^B^	Treatment	Diagnosis^C^
Breault et al. 1997 [13]	M, 76	Mandible, Incisor	No	SCTG	15	Excision	Cyst
Harris 2002 [14]	F, 27	Mandible, Canine	Yes	SCTG	13	Punch biopsy, Gingivoplasty	Cyst
Wei and Geivelis 2003 [15]	F, 40	Mandible, Incisor	Yes	SCTG, Gingivoplasty	9	Incision, Gingivoplasty, Free SCTG	NA
de Castro et al. 2007 [16]	F, 22	Mandible, Canine	Yes	FGG	11	Excision, FGG	Cyst
Fletcher et al. 2011 [17]	F, 45	Maxilla, Canine (implant)	No	SCTG (multiple)	48	Excision, Bone allograft	Cyst
Present report	M, 46	Mandible, Canine	No	SCTG	0.75	Excision	Cyst

^A^Patient gender (female, male) and age (in years). ^B^Time (in months) between preceding treatment and clinical appearance of lesion. ^C^Reported histopathologic diagnosis. NA: not available.
